# Phylogeny and Expression Atlas of the *NITRATE TRANSPORTER 1/PEPTIDE TRANSPORTER FAMILY* in Agave

**DOI:** 10.3390/plants11111434

**Published:** 2022-05-27

**Authors:** Shibei Tan, Yanqiong Liang, Yanlei Huang, Jingen Xi, Xing Huang, Xiaohan Yang, Kexian Yi

**Affiliations:** 1 Environment and Plant Protection Institute, Chinese Academy of Tropical Agricultural Sciences, Haikou 571101, China; tanshibei915@163.com (S.T.); yanqiongliang@126.com (Y.L.); xijingen@163.com (J.X.); 2 College of Plant Science and Technology, Huazhong Agricultural University, Wuhan 430070, China; hyl4532323642022@163.com; 3 Biosciences Division, Oak Ridge National Laboratory, Oak Ridge, TN 37831, USA; 4 Key Laboratory of Integrated Pest Management on Tropical Crops, Ministry of Agriculture and Rural Affairs, Haikou 571101, China; 5 Hainan Key Laboratory for Monitoring and Control of Tropical Agricultural Pests, Haikou 571101, China

**Keywords:** nitrate transporter, *NPF*, phylogeny, gene expression, agave

## Abstract

*Agave* species are widely cultivated crassulacean acid metabolism (CAM) plants for alcoholic beverages, food and fiber production. Among these, the *Agave* hybrid H11648 ((*A. amaniensis* × *A. angustifolia*) × *A. amaniensis*) is the main cultivar for sisal fiber in the tropical areas of Brazil, China, and African countries. The plants of *Agave* hybrid H11648 have a long life cycle and large leaves, which require a huge amount of nitrogen nutrient. However, the molecular basis of nitrogen transport and allocation has not been well understood in agave. In this study, we identified 19 *NITRATE TRANSPORTER 1*/*PEPTIDE TRANSPORTER FAMILY*(*NPF*) genes (called *AhNPFs*) with full-length coding sequences in *Agave* hybrid H11648. Our analysis of gene expression in various types of tissues revealed the tissue-specific expression pattern of *AhNPFs*. We further examined their expression patterns at different leaf developmental stages, under abiotic/biotic stresses and nutrient deficiency. The results reveal several candidate regulators in the agave *NPF* family, including *AhNPF4.3*/*5.2*/*7.1.* We first characterized the *NPF* genes in agave based on published leaf transcriptome datasets and emphasized their potential functions. The study will benefit future studies related to nitrogen nutrient in agave.

## 1. Introduction

Nitrate is one of the major nitrogen resources for plant growth and development [1]. As one of the most important nitrate transporters, *NITRATE TRANSPORTER 1*/*PEPTIDE TRANSPORTER FAMILY* (*NPF*) has been well functionally characterized well in arabidopsis (*Arabidopsis thaliana* L.) [2]. The arabidopsis genome contains 53 *NPF* genes, which are clustered into eight clades. Among these, *AtNPF1.1* (*NRT1.12*) and *AtNPF1.2* (*NRT1.11*) mediate nitrate allocation to young leaves [3]. *AtNPF2.3* contributes to nitrate translocation to shoots under salt stress [4]. *AtNPF2.7* regulates nitrate efflux in roots under acidic conditions [5]. *AtNPF2.9* (*NRT1.9*) plays important roles in the phloem loading of nitrate in roots [6]. *AtNPF2.12* (*NRT1.6*) modulates nitrate transport to the embryo [7]. The phloem-expressed *AtNPF2.13* (*NRT1.7*) is responsible for source-to-sink remobilization of nitrate [8]. *AtNPF3.1* modulates nitrite accumulation in leaves [9]. *AtNPF4.6* (*NRT1.2*/*AIT1*) controls nitrite uptake in roots [10]. *AtNPF5.5* affects nitrogen accumulation in the embryo [11]. *AtNPF5.11*, together with *AtNPF5.12* and *AtNPF5.16*, is involved in vacuolar nitrate efflux and reallocation [12]. *AtNPF6.2* (*NRT1.4*) alters leaf development by nitrate storage in the petiole [13]. *AtNPF6.3* (*NRT1.1*) regulates lateral root growth by nitrate-regulated auxin transport [14]. *AtNPF7.2* (*NRT1.8*) functions in nitrate removal from the xylem sap [15]. *AtNPF7.3* (*NRT1.5*) is related to root-to-shoot nitrate transport [16]. *AtNPF8.1* (*PTR1*) and *AtNPF8.2* (*PTR5*) transport dipeptides in root and pollen [17]. Additionally, several *AtNPFs* have the functions of transporting plant hormones, including auxin, abscisic acid and gibberellin [18]. In contrast, there are 93 *NPF* genes in the rice (*Oryza sativa* L.) genome with 7 of them having functions related to nitrate transport and allocation, including *OsNPF2.2* (*OsPTR2*), *OsNPF2.4*, *OsNPF6.5* (*OsNRT1.3*/*NRT1.1B*), *OsNPF7.2*, *OsNPF7.3* (*OsPTR6*), *OsNPF8.9* (*OsNRT1*) and *OsNPF8.20* (*OsPTR9*) [2].

*Agave* species, which perform crassulacean acid metabolism (CAM) photosynthesis, are widely used for the production of alcoholic beverages, food and fiber [19]. Agave plants have great potential for producing bioenergy in arid and semi-arid regions, with a large nitrogen demand during the life cycle [20,21]. However, the molecular basis of nitrogen transport and allocation has not been well studied in agave [22]. In China, the most widely cultivated *Agave* species is *Agave* hybrid H11648 ((*A. amaniensis* × *A. angustifolia*) × *A. amaniensis*), with a main purpose of sisal fiber production [23]. It is of great importance to reveal the mechanism of nitrogen transport and allocation in the leaf, which is both a vegetative and harvestable organ. It is very challenging to sequence and assemble the large agave genomes [24]. *Agave* hybrid H11648 has been proved to have a high tolerance to heavy metals such as copper (Cu) and lead (Pb) [23]. Additionally, there are two main abiotic/biotic stresses during agave cultivation, including chill and zebra disease caused by *Phytophthora nicotianae* Breda [19]. Based on the published leaf transcriptome data of *Agave* H11648, we identified *NPF* genes in this agave species [25]. We also examined the expression profiles of *AhNPFs* at different leaf developmental stages, under abiotic/biotic stresses, and after nutrient deficiency treatments. Our findings will provide a guideline for characterizing the candidate functions of *AhNPFs* in leaf development and multi stress responses [26,27]. 

## 2. Results

### 2.1. Identification and Subcellular Localization of Agave NPF Genes

All arabidopsis and rice *NPF* genes were selected as query to search against asparagus (*Asparagus officinalis* L.) genome and agave transcriptome datasets [25,28,29]. Asparagus was selected as a reference due to its close phylogenetic relationship with *Agave* species [28]. As a result, 53 and 19 *NPF* genes were identified in asparagus and agave, respectively (Table 1, Table S1). In agave, the lengths of the coding sequences ranged from 1377-2400 bp, with predicted proteins of 458-799 aa. The molecular weights were from 50,193.55 to 89,795.73 and theoretical isoelectric points (pI) were from 5.77 to 9.29. All the agave *NPF* genes were predicted to be localized at the plasma membrane (Table 1). Additionally, these proteins contained at least six transmembrane helices at the N- or C-termini (Figure S1).

### 2.2. Phylogenetic Analysis of Agave NPF Genes

**Figure 1:**
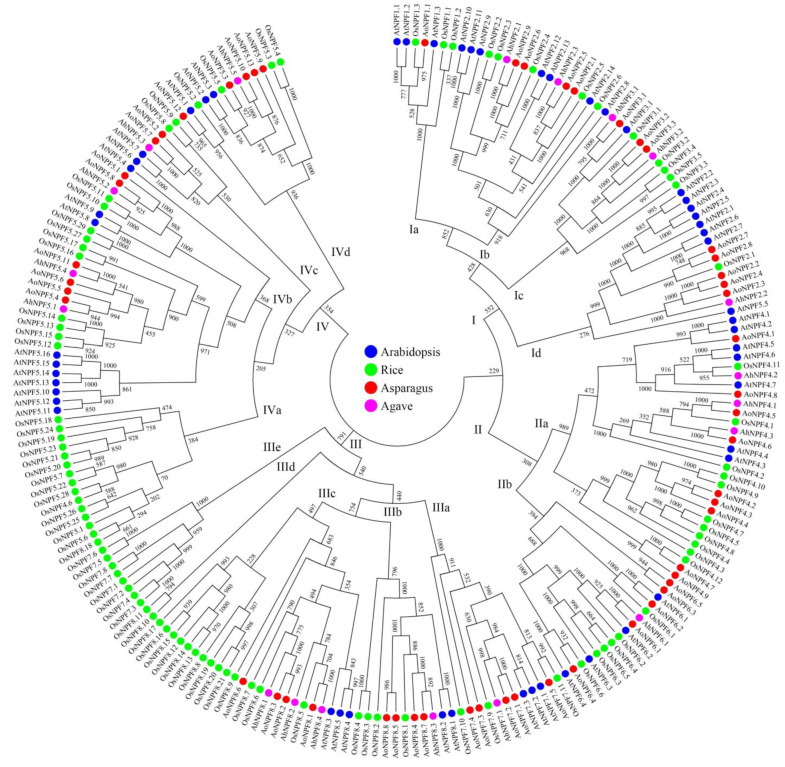
Phylogenetic analysis of *NPF* family. The protein names of arabidopsis, rice, asparagus and agave are labeled in blue, green, red and pink, respectively.

The NPF protein sequences of arabidopsis (53), rice (93), asparagus (53) and agave (19) were selected for phylogenetic analysis. These proteins were clustered into four groups (Figure 1). Group I was further divided into four subgroups, including one NPF1 subgroup, one NPF3 subgroup and two NPF2 subgroups. Group II was divided into two subgroups, containing NPF4 and NPF6 proteins, respectively. There were five subgroups in group III, including one NPF7 subgroup and four NPF8 groups. All NPF5 proteins were clustered into group IV except *AtNPF5.5*. There were also four subgroups containing only rice NPF proteins, including subgroups IIIb, IIIc, IIId and IVa.

### 2.3. Expression Profiles of AhNPFs in Different Agave Tissues

**Figure 2:**
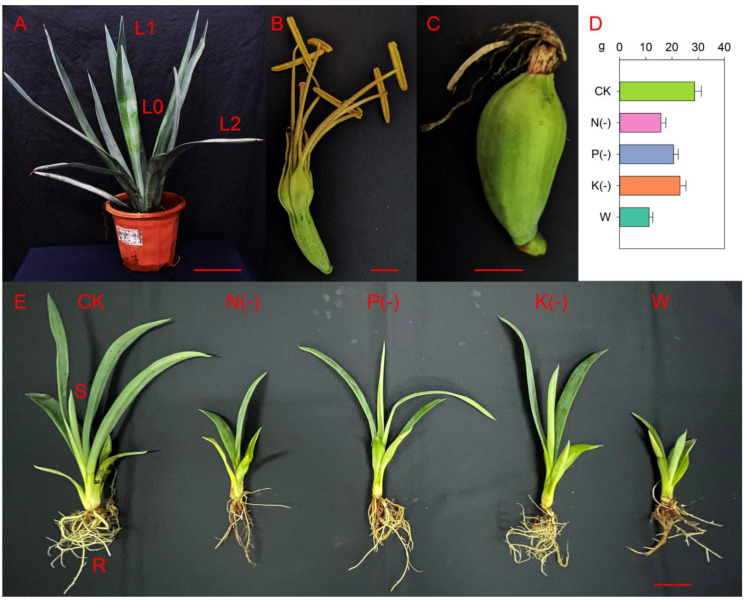
Photographs of agave plants (**A**, bar = 10 cm), flower (**B**, bar = 1 cm), fruit (**C**, bar = 1 cm), nutrient deficiency-induced biomass (**D**) and morphological features (**E**, bar = 5 cm) of agave seedlings. L0, L1 and L2 represent shoot, unexpanded leaf and expanded leaf in potted agave plants, respectively. CK, N(-), P(-), K(-) and W in D and E represent that the agave seedlings were cultured with full-, nitrogen free-, phosphorus free-, potassium free-Hoagland nutrient solutions and water, respectively. Shoot (S) and root (R) of agave seedlings are shown in (**E**).

**Figure 3:**
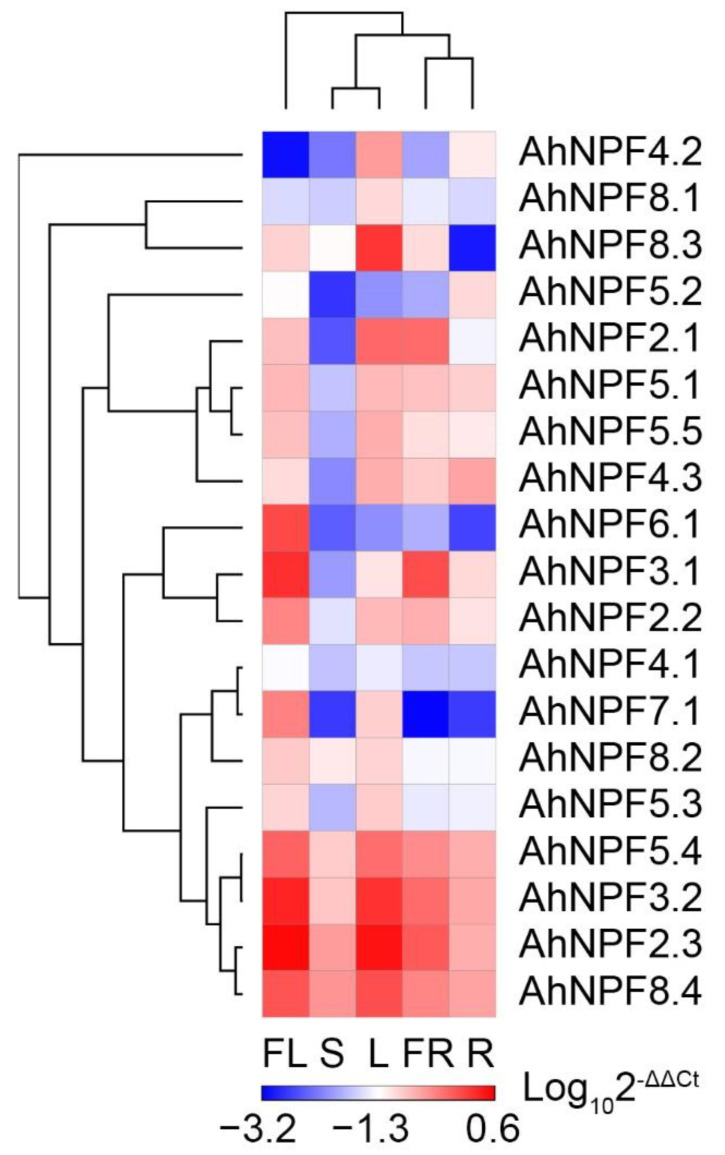
Expression pattern of agave *NPF* genes in different tissues by qRT-PCR. FL, S, L, FR and R represent flower, shoot, leaf, fruit and root, respectively. The genes and tissues were clustered by hierarchical clustering method [30].

The expression of *NPF* genes was examined in five agave tissues, including the flower, shoot, leaf, fruit and root (Figure 2). Most *AhNPFs* showed high expression in at least one tissues (Figure 3). There were eight *AhNPFs* showing high expression in flower and leaf tissues, including *AhNPF2.3*/*3.2*/*4.1*/*5.3*/*5.4*/*7.1*/*8.2*/*8.4*. We found that *AhNPF2.2*/*6.1*, *AhNPF4.2*/*5.5*/*8.1*/*8.3* and *AhNPF5.2* were highly expressed in the flower, leaf and root, respectively. Also, we found high expression of *AhNPF2.1* in the leaf/fruit and *AhNPF3.1* in the flower/fruit. *AhNPF4.3* and *AhNPF5.1* showed high expression in most tissues except the shoot.

### 2.4. Expression Profiles of AhNPFs during Leaf Development

**Figure 4:**
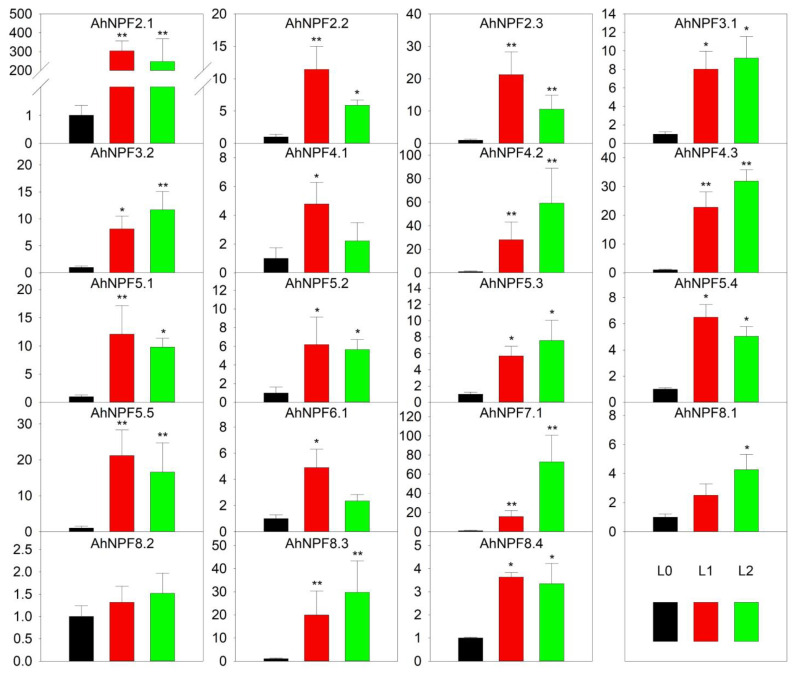
Expression pattern of agave *NPF* genes at leaf developmental stages by qRT-PCR. Y-axis represents relative expression level. L0, L1 and L2 of x-axis represent shoot, unexpanded leaf and expanded leaf, respectively. The error bar represents the standard error. * and ** represent that expression level was increased or decreased by more than 3-fold and 10-fold compared with L0, respectively. The expression values of L0 stages were normalized as 1.

We have also examined the expression patterns of *AhNPFs* at different developmental stages of the agave leaf (Figure 2). The results revealed that most *AhNPFs* were significantly up-regulated at least in the unexpanded (L1) or expanded (L2) leaf except *AhNPF8.2*, when compared with the shoot stage (L0) (Figure 4). Among these, there were eight and nine *AhNPFs* with expression levels increased by more than 3- and 10-fold at the L1 stage, respectively. The expressions of 16 *AhNPFs* were increased more than 3-fold and eight were over 10-fold when comparing L2 with L0. Additionally, only the *AhNPF7.1* changed expression levels more than 3-fold when comparing L2 with L1.

### 2.5. Expression Profiles of AhNPFs under Abiotic/Biotic Stresses

**Figure 5:**
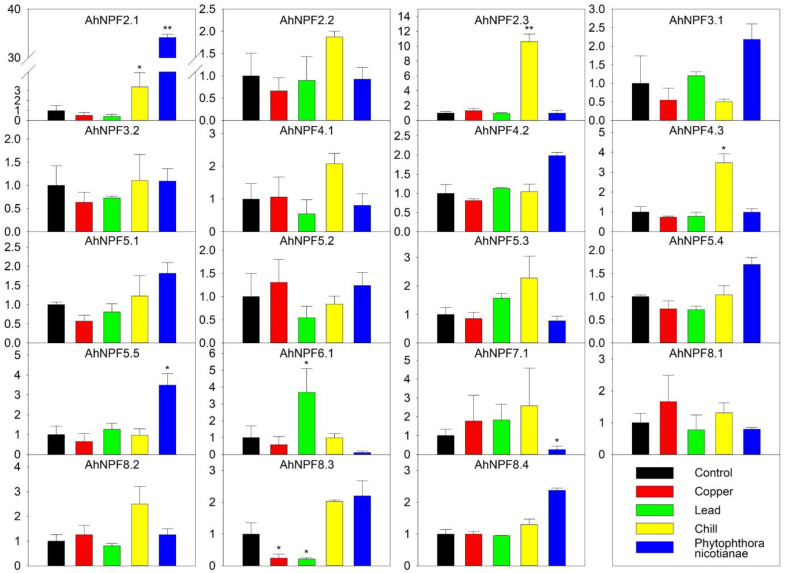
Expression pattern of agave *NPF* genes under abiotic/biotic stresses by qRT-PCR. Y-axis represents relative expression level. The labels of x-axis represent control, CuSO_4_ treatment, Pb(NO_3_)_2_ treatment, chill treatment and *Phytophthora nicotianae* Breda inoculation, respectively. The error bar represents the standard error. * and ** represent that expression level was increased or decreased by more than 3-fold and 10-fold compared with control, respectively. The expression values of control were normalized as 1.

We also evaluated the expression of *AhNPFs* in response to five abiotic/biotic stress treatments, including treatments of copper salt, lead salt, chill and *Phytophthora nicotianae* Breda inoculation (Figure 5). As a result, 12 *AhNPFs* showed moderate expressions under different stresses. *AhNPF8.3* was the only gene with a changed expression level over 3-fold under copper stress. The expression of *AhNPF6.1* and *AhNPF8.3* were increased and decreased over 3-fold under lead stress, respectively. There were three *AhNPFs* up-regulated over 3-fold under chill stress, including *AhNPF2.1*/*2.3*/*4.3*. Additionally, *AhNPF2.1*/*AhNPF8.2* and *AhNPF7.1* were up- and down-regulated over 3-fold under the biotic stress, respectively.

### 2.6. Expression Profiles of AhNPFs under Nutrient Deficiency

**Figure 6:**
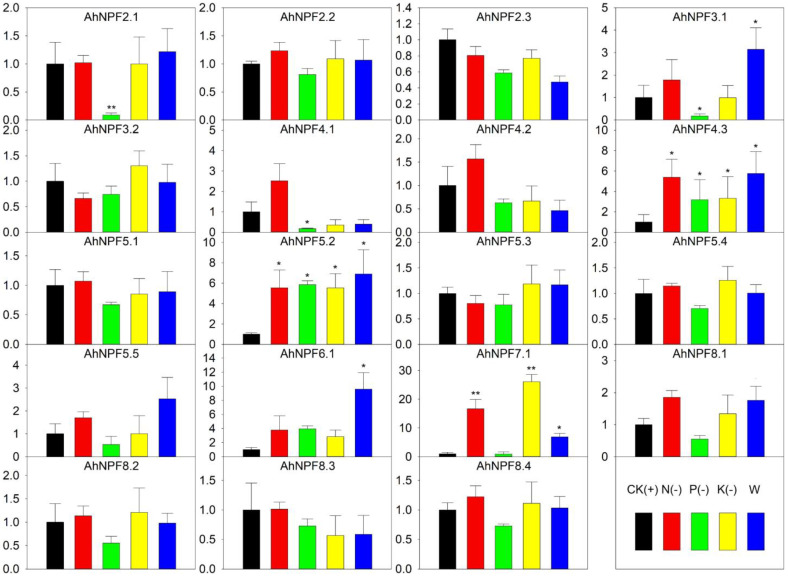
Expression pattern of agave *NPF* genes under nutrient deficiency by qRT-PCR. Y-axis represents relative expression level. CK, N(-), P(-), K(-) and W of x-axis represent that the agave seedlings were cultured with full-, nitrogen free-, phosphorus free-, potassium free-Hoagland nutrient solutions and water, respectively. The error bar represents the standard error. * and ** represent that expression level was increased or decreased by more than 3-fold and 10-fold compared with CK, respectively. The expression values of control were normalized as 1.

We further carried out nutrient deficiency treatments to evaluate the expression patterns of *AhNPFs*. The full Hoagland nutrient solution was selected as control (CK). Hoagland nutrient solutions without nitrogen (N-), phosphorus (P-), potassium (K-) and water (W) were set as treatments. The biomass and growth were significantly restricted by the four treatments (Figure 2). The result indicated that 12 *AhNPFs* were less sensitive to nutrient deficiency (Figure 6). *AhNPF4.3* and *AhNPF5.2* increased their expressions over 3-fold under N-, P-, K- and W. *AhNPF7.1* was up-regulated over 3-fold under N-, K- and W. The expressions of three *AhNPFs* (*AhNPF2.1*/*3.1*/*4.1*) decreased over 3-fold under P-. Additionally, there were another two *AhNPFs* (*AhNPF3.1*/*6.1)* that showed up-regulated expressions over 3-fold in W.

## 3. Discussion

### 3.1. Identification of Agave NPF Genes

In the present study, we have successfully identified 19 *NPF* genes according to a previous transcriptome dataset, which was a relatively small number compared with asparagus and other species (Table 2). This might be caused by tissue-specific expression because the leaf was the only tissue used for transcriptome assembly in the previous study [25]. It is possible that more agave *NPF* genes will be identified based on the agave genome sequence which is not available yet. The other agave *NPF* genes might have an extremely low expression in the leaf, which made it hard to assemble the full-length transcript sequence from Illumina sequencing data [31]. The PacBio SMRT (single molecule real-time) sequencing technology is very powerful for detecting genes with low expressions [32]. There was a similar expression pattern of *NPF* genes in maize (*Zea mays* L.) and more than a half of 79 *NPF* genes showed no expressions in the leaf [29,33]. Most agave *NPF* genes in this study did not show obvious specific expression in single tissues (Figure 3). Most *AhNPFs* showed relatively high expressions in no less than two tissues. This might be related to the widely spread transport system of nitrogen, which is important for all of the growth and development processes in plants [2]. There are other factors that might affect gene function and expression, such as nucleotide sequence changes by chromosome recombination [34]. The large amount of 60 chromosomes in *A*. H11648 has significantly improved the frequency of chromosome recombination compared with the 10 chromosomes in asparagus [24,28]. Insertion or deletion in the promoter and coding region may be introduced, which would directly alter the function and expression of genes [35,36]. The species-specific expansion of *NPF* genes also exists in subgroup IIIe and IVa in rice (Figure 1). Further studies are still needed to reveal the evolution patterns of *NPF* genes in plants.

### 3.2. Regulation of Agave NPF Genes Associated with Leaf Development, Abiotic/Biotic Stresses and Nutrient Deficiency

We examined the expression patterns of *AhNPFs* in different tissues, during the process of leaf development and under abiotic/biotic stresses and nutrient deficiency. The results indicate their diverse functions in the growth and development of agave. Each *AhNPF* gene was dominantly expressed in one or more agave tissues, which indicates their tissue-specific expression patterns. Additionally, the relatively high expression patterns of *AhNPF2.3*/*3.2*/*5.4*/*8.4* reveal their importance in the basic nitrogen transport system in agave tissues [2]. Most Agave *NPF* genes were significantly up-regulated during leaf development, which is consistent with the expression pattern of their orthologous genes in maize [33]. The tiny young shoot is much smaller than the large leaf and its total demand of nitrogen is also much less than the leaf [37]. Additionally, more than half of the *NPF* genes (47) showed no or extremely low expression levels in the shoot and leaf tissues of maize, which also provided a possible reason for the relatively small amount of *NPF* genes identified in the agave leaf transcriptome [33]. The expression analysis has also revealed the numbers of candidate *AhNPFs* under copper, lead, chill and fungus stress. There are one, two, three and three *AhNPFs* significantly up- or down-regulated under the four stresses, respectively (Figure 5). The results indicate that *AhNPFs* had different responsesto abiotic/biotic stresses. This might be caused by different sensing and regulating networks upstream of various antioxidant enzymes, which are commonly produced to scavenge reactive oxygen species triggered by stresses in plants [38]. The transport and allocation of nitrate is necessary for the synthesis of these enzymes [26]. Moreover, the expression analysis also indicated the candidate nitrate transporters in agave, including *AhNPF4.3*/*5.2*/*7.1* (Figure 6). Interestingly, they are also involved in P and K responses. Besides, *AhNPF2.1*/*3.1*/*4.1* are only involved in the P response and do not have obvious responses to N and K. We infer the existence of crosstalk among the three nutrients, which was also reported in *Brassica napus* [27,39]. However, more evidence is still needed to reveal the interactions among N, P and K nutrients, which will contribute to the rational application of fertilizers in agave.

## 4. Materials and Methods

### 4.1. Sequence Retrieval and Phylogenetic Analysis

The NPF proteins of arabidopsis (53) and rice (93) were selected as the query for homologous sequence identification by the TBlastn method [29,40]. The transcriptome of *Agave*. hybrid H11648 was selected for sequence retrieval and asparagus was selected as a closely related reference [25,28]. The ORF-FINDER software was used to identify agave *NPF* genes with full coding sequences, which were further selected for subcellular localization prediction using the CELLO software [41,42]. The protein sequences of arabidopsis, rice, asparagus and agave were selected for phylogenetic analysis. The phylogenetic tree was constructed with the neighbor-joining method and bootstrap values tested for 1000 trials by the MEGA 5.0 software [43].

### 4.2. Plant Materials and RNA Extraction

The samples of the leaf, flower and fruit were collected from a flowering plant of *Agave* hybrid H11648at the germplasm garden of Guangxi Subtropical Crops Research Institute (22.90° N, 108.33° E). Fruit samples were collected one month after flowering. The other tissue samples were collected at the Environment and Plant Protection Institute, Chinese Academy of Tropical Agricultural Sciences (19.99° N, 110.33° E). Shoot and root samples were collected from seedlings after one month of being cultured in Hoagland nutrient solution [44]. The samples of leaf development, abiotic and biotic stresses were collected according to a previous study [23]. The details were as follows. The shoot, unexpanded leaf and fully expanded leaf were separately collected as different developmental stages from 2-year-old plants. The stress treatments were carried out with agave seedlings planted in pots. The leaf samples were collected at 2 weeks after watering with solutions of CuSO_4_ (1 g/Kg) and Pb(NO_3_)_2_ (1.3 g/Kg), and 5 days after the inoculation of *Phytophthora nicotianae* Breda. The potted seedlings were placed in an incubator at 6 °C as chill treatment and sampled at 24 h. Untreated leaves were also sampled as control. The agave seedlings were cultured in water for rooting and then utilized for nutrient deficiency treatments after one week. The full Hoagland nutrient solution was selected as control [44]. The solutions without N, P and K nutrients were selected as treatments [45]. Water was selected as a nutrient-free treatment. The leaves were sampled after the seedlings were cultured for 6 months. Each sample or treatment was repeated three times as biological replicates. All the samples were ground into powder after immediate freezing in liquid nitrogen. Total RNA was extracted with the Tiangen RNA prep Pure Plant Kit (Tiangen Biomart, Beijing, China) and stored at −80 °C.

### 4.3. Analysis of Gene Expression Using Quantitative Reverse Transcription PCR (qRT-PCR)

The total RNA was reverse transcribed into cDNA for qRT-PCR analysis by the GoScript Reverse Transcription System (Promega, Madison, WI, USA). Each qRT-PCR reaction solution was mixed to a final volume of 20 μL, including 10 μL of TransStart Tip Green qPCR Supermix (Transgen Biotech, Beijing, China), 0.4 μL of Passive Reference Dye (50×) (Transgen Biotech, Beijing, China), 1 μL of cDNA template, 0.5 μL of 2 gene-specific primers (10 μM) and 7.6 μL ddH_2_O. The QuantStudio 6 Flex Real-Time PCR System (Thermo Fisher Scientific, Waltham, MA, USA) was used for qRT-PCR reaction with a program including 94 °C for 30 s, 40 cycles of 94 °C for 5 s and 60 °C for 30 s, and a final dissociation stage. The reaction was carried out in each sample 3 times as the technical repeat. Gene specific primers of *AhNPFs* were designed by the Primer 3 software (Table 2) [46]. The *protein phosphatase 2A* (*PP2A*) gene was used as the endogenous control [23]. We selected the ΔΔCt method to calculate relative expression levels, as reported in a previous study [47].

## 5. Conclusions

In this study, we identified 19 *NPF* genes on the leaf transcriptome of *Agave* hybrid H11648. Our analysis of gene expression in various types of tissues revealed the tissue-specific expression pattern of *AhNPFs*. We further examined their expression patterns at different leaf developmental stages, under abiotic/biotic stresses and nutrient deficiency. The results reveal several candidate regulators in the agave *NPF* family, including *AhNPF4.3*/*5.2*/*7.1.* Our results provide a guideline to reveal the complex genetic dissection of the agave *NPF* family, which will benefit future studies related to the mechanism of nitrate nutrients in agave.

## Figures and Tables

**Table 1:** Gene ID, accession, coding sequence length, molecular weight, theoretical isoelectric point (pI) and subcellular localization of agave *NPFs*.

ID	Accession	Coding Sequence (bp)	Predicted Protein (aa)	Molecular Weight	pI	Subcellular Localization
*AhNPF2.1*	DN41135_c0_g1_i1	1767	588	65,558.83	8.91	PlasmaMembrane (4.946)
*AhNPF2.2*	DN48816_c0_g1_i2	2400	799	89,795.73	9.29	PlasmaMembrane (3.655)
*AhNPF2.3*	DN50815_c0_g2_i3	1866	621	68,836.88	8.57	PlasmaMembrane (4.952)
*AhNPF3.1*	DN41985_c0_g1_i1	1797	598	66,083.81	8.58	PlasmaMembrane (4.828)
*AhNPF3.2*	DN49643_c0_g1_i2	1788	595	65,163.86	9.11	PlasmaMembrane (4.727)
*AhNPF4.1*	DN35785_c0_g1_i1	1761	586	64,956.66	8.90	PlasmaMembrane (4.906)
*AhNPF4.2*	DN44495_c0_g1_i3	1743	580	63,224.67	8.98	PlasmaMembrane (4.860)
*AhNPF4.3*	DN46864_c0_g1_i1	1782	593	66,186.76	8.21	PlasmaMembrane (4.872)
*AhNPF5.1*	DN41592_c0_g1_i2	1668	555	61,626.22	5.80	PlasmaMembrane (4.911)
*AhNPF5.2*	DN44315_c0_g1_i1	1611	536	59,762.91	6.47	PlasmaMembrane (4.969)
*AhNPF5.3*	DN46789_c0_g1_i1	1602	533	59,330.15	8.77	PlasmaMembrane (4.937)
*AhNPF5.4*	DN47034_c0_g2_i1	1734	577	63,963.40	8.70	PlasmaMembrane (4.971)
*AhNPF5.5*	DN48393_c3_g3_i2	1761	586	65,431.38	9.05	PlasmaMembrane (4.836)
*AhNPF6.1*	DN42911_c0_g2_i1	1737	578	62,801.28	9.00	PlasmaMembrane (4.919)
*AhNPF7.1*	DN45587_c0_g2_i1	1854	617	68,530.90	5.77	PlasmaMembrane (4.917)
*AhNPF8.1*	DN41583_c0_g1_i1	1377	458	50,193.55	6.82	PlasmaMembrane (4.782)
*AhNPF8.2*	DN43232_c0_g2_i2	1773	590	65,292.70	5.87	PlasmaMembrane (4.876)
*AhNPF8.3*	DN50692_c0_g1_i8	1701	566	62,878.70	7.09	PlasmaMembrane (4.754)
*AhNPF8.4*	DN51386_c0_g1_i1	1758	585	64,615.74	6.18	PlasmaMembrane (4.838)

**Table 2:** Primers used for qRT-PCR analysis.

ID	Forward Primer	Reverse Primer	Product Size (bp)
*AhNPF2.1*	GCGCAGACCAATTCAATCCT	CGTGCCGATGAAGAAGAAGG	206
*AhNPF2.2*	TCTCCTTCTTCCAAGCCCTG	AGCTCACCTGATAGACACCG	160
*AhNPF2.3*	GCTTCCAAATTCCTCCTGCC	CACAATCATGGACAGAGCCG	182
*AhNPF3.1*	CCTCGTTGCTGGATTCATCG	CTCCCCGCTGATATCACCAT	240
*AhNPF3.2*	TGCTCTAATCGCCGACTCAT	ACCAATGCAAGTCAGGAGGA	208
*AhNPF4.1*	ATCTGCCCGTCAAACTCTGA	CTTGCTCAACCTGTGTCACC	181
*AhNPF4.2*	TCATCCCTGAAGCCTCCATC	GTGTCTTCGTCGAACTGCTC	173
*AhNPF4.3*	TGGTGGAGAGCAAGAGAAGG	ACCCGAATGAGTACGAGCAA	201
*AhNPF5.1*	ATCCTCCATTTTGCCAAGCG	GACCAGAACCGAGACCATCA	223
*AhNPF5.2*	TGGCTGGTGTGGAGAGATTT	CAAGTCCCGCAACATAGAGC	222
*AhNPF5.3*	ACCAACACCTGCGACAAATC	CTCCACTACAGAGGCCACAA	199
*AhNPF5.4*	ATTGCGAGCGAGACCAAAAG	TCTCAGCAACCTCAACACCT	187
*AhNPF5.5*	AGATAACGTGGGGTGGACTG	AGCTCCTTGGGGTCATTAGG	195
*AhNPF6.1*	CAATTCGACGACAAGGACCC	AGGACCATCGATATGCAGCA	170
*AhNPF7.1*	ACCTGGGCTCTCTCTTTTCC	TTGACCGTCCATTTCCTCGA	214
*AhNPF8.1*	ATCAAACGCCTCCAGATTGC	CGTCGTCACAAGAGCAAGAG	204
*AhNPF8.2*	GCAAGATAACTGCGGATGGG	AGAGAACTGTCAAGAGGGGC	195
*AhNPF8.3*	GCATAATCTTCGCCACGGTT	CTCATGTCCGGTGAACTTGC	200
*AhNPF8.4*	CCGCAGTCTACAGCCAAATC	AAGCCTCTCTCTTTGCCAGT	193
*PP2A*	CCTCCTCCTCCTTCGGTTTG	GCCATGAATGTCACCGCAGA	235

## Data Availability

All data are contained within the article or Supplementary Materials.

## Supplementary Materials

The following supporting information can be downloaded at: https://www.mdpi.com/article/10.3390/plants11111434/s1, Figure S1: Transmembrane topology analysis of agave NPF proteins.; Table S1: The *NPF* gene family in asparagus.
