# Supplementary figures and images for: Phylogeny and Expression Atlas of the NITRATE TRANSPORTER 1/PEPTIDE TRANSPORTER FAMILY in Agave
Source: Plants (Basel). 2022 May 27;11(11):1434. doi: 10.3390/plants11111434 (PMC9182991; doi:10.3390/plants11111434)

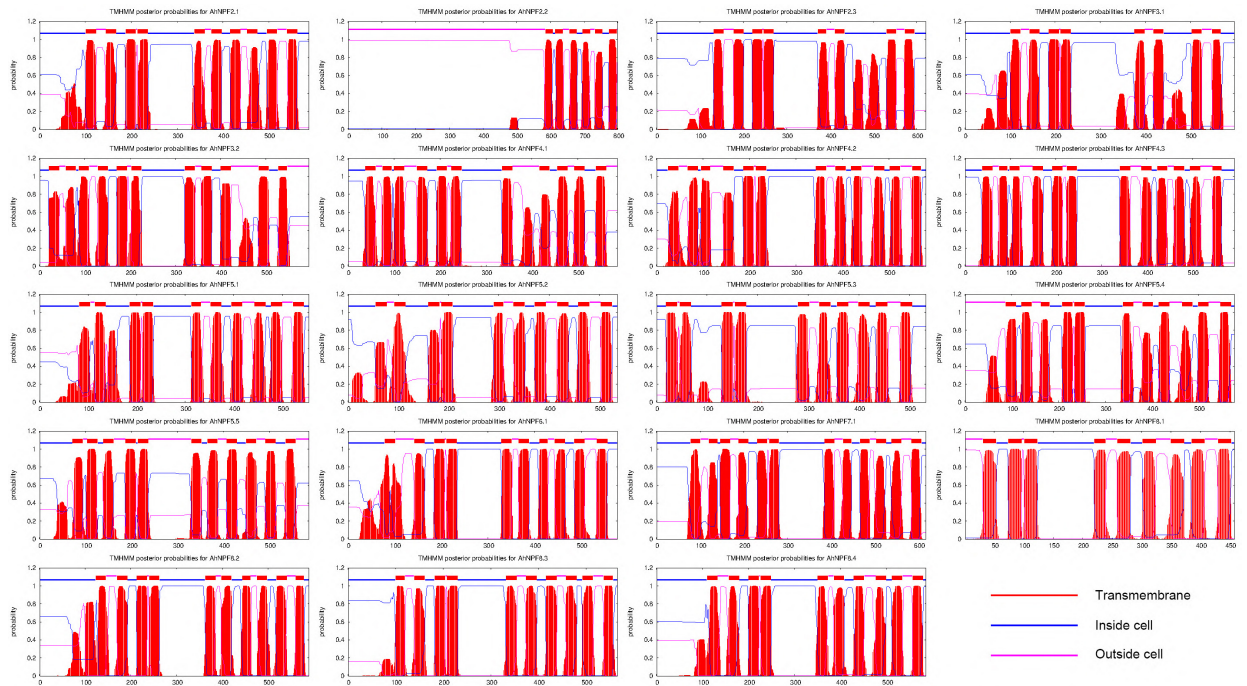

Figure S1. Transmembrane topology analysis of agave NPF proteins.

Supplement: Supplementary file 1 [file plants-11-01434-s001.zip › plants-1729665-Figure S1.pdf]
